# Symptoms of Posttraumatic Stress after Intensive Care Delirium

**DOI:** 10.1155/2015/876947

**Published:** 2015-10-18

**Authors:** Helle Svenningsen, Ingrid Egerod, Doris Christensen, Else Kirstine Tønnesen, Morten Frydenberg, Poul Videbech

**Affiliations:** ^1^Department of Nursing, Faculty of Health Sciences, VIA University College, Hedeager 2, 8200 Aarhus N, Denmark; ^2^Copenhagen University Hospital Rigshospitalet, Trauma Center 3193, Blegdamsvej 9, 2100 Copenhagen Ø, Denmark; ^3^Department of Anaesthesia, Hillerød Hospital, Dyrehavevej 29, 3400 Hillerød, Denmark; ^4^Department of Anaesthesia and Intensive Care Medicine, Aarhus University Hospital, Building 21, 1, Nørrebrogade 44, 8000 Aarhus C, Denmark; ^5^Department of Biostatistics, Institute of Public Health, Aarhus University, Building 1261, Room 221, Bartholins Allé 2, 8000 Aarhus, Denmark; ^6^Department Q, Aarhus University Hospital, Risskov Skovagervej 2, 8240 Risskov, Denmark

## Abstract

*Introduction*. Long-term psychological consequences of critical illness are receiving more attention in recent years. The aim of our study was to assess the correlation of ICU-delirium and symptoms of posttraumatic stress disorder (PTSD) anxiety and depression after ICU-discharge in a Danish cohort.* Methods. *A prospective observational cohort study assessing the incidence of delirium in the ICU. Psychometrics were screened by validated tools in structured telephone interviews after 2 months (*n* = 297) and 6 months (*n* = 248) after ICU-discharge.* Results*. Delirium was detected in 54% of patients in the ICU and symptoms of PTSD in 8% (2 months) and 6% (6 months) after ICU-discharge. Recall of ICU stay was present in 93%. Associations between ICU-delirium and post-discharge PTSD-symptoms were weak and insignificant. Memories of delusions were significantly associated with anxiety after two months. Remaining associations between types of ICU-memories and prevalence of post-discharge symptoms of PTSD, anxiety, and depression were insignificant after adjusting for age. Incidence of ICU-delirium was unaffected by preadmission use of psychotropic drugs. Prevalence of PTSD-symptoms was unaffected by use of antipsychotics and sedation in the ICU.* Conclusion.* ICU-delirium did not increase the risk of PTSD-symptoms at 2 and 6 months after ICU discharge.

## 1. Introduction

Each year more people worldwide become critically ill and require treatment in the intensive care unit (ICU). Apart from the disease itself, the ICU environment exposes patients to extreme stressors, including painful procedures, multiple medications, mechanical ventilation, and inability to communicate. It has been demonstrated that most ICU patients experience episodes of delirium [1–3], characterized by a disturbance of consciousness (i.e., reduced clarity of awareness of the environment) with reduced ability to focus, sustain, or shift attention. A change in cognition or the development of a perceptual disturbance that is not better accounted for by a preexisting, established or evolving dementia.

The disturbance develops over a short period of time (usually hours to days) and tends to fluctuate during the course of the day, and there must be evidence from the history, physical examination, or laboratory findings that the disturbance is caused by the direct physiological consequences of a general medical condition [4]. Hallucinations, delusions, and paranoia are common but do not necessarily emerge in delirium. In Danish ICUs, at least one episode of delirium has been detected in 65% of the patients with a stay of more than 48 hours, and though very light or no sedation only 9% of positive delirium scores were in hyperactive patients [5], so even very scary delusions do not necessarily lead to hyperactive delirium. This leaves us with no easy guide to how patients are mentally after ICU-delirium. Does the memoires of the delirium and its often scary and bizarre word lead to mental disorders or symptoms of posttraumatic stress disorder (PTSD) in post-ICU patients? In systematic reviews, the prevalence of PTSD-symptoms in ICU survivors was as high as 64% with a mean of 29% [6], though 9% to 27% in the years 2008–2012 [7]. The prevalence varies, however, according to country, assessment tool, and treatment and care regimes.

## 2. Aims of the Study

The primary aim of our study was to assess the correlation between ICU-delirium and the prevalence of PTSD-symptoms, other anxiety and depressive symptoms at 2 and 6 months after discharge from ICU. We also wished to assess whether prior mental illness expressed by number of redeemed prescriptions for psychotropic drugs prior to admission was associated with development of ICU-delirium. Finally, we wished to explore the associations between post-ICU PTSD-symptoms, anxiety, and depression.

## 3. Materials and Methods

### 3.1. Sample

Adult patients admitted to three mixed ICUs at two university hospitals in Denmark were consecutively included in this prospective observational study. Inclusion criteria were ICU stay >48 hours, age >17 years, and ability to communicate in Danish. Patients were excluded in cases of severe brain damage restricting communication, or PTSD diagnosed prior to admission. Informed consent was obtained during the first week after ICU-discharge and rereached at the start of the structured telephone interviews performed by the first author after 2 and 6 months. Patients' answers were entered to a database as soon as they appeared. Before each call, the national database was checked to minimize the risk of bothering relatives if the patient had died in the meantime. Patients were called up to five times to make contact. If there was still no contact, the patient was excluded. Patients were excluded from the second interview if they were readmitted to ICU after the first interview.

### 3.2. Background Measures

Demographics, severity of illness during the first 24 hours of ICU-admission using Simplified Acute Physiology Score (SAPSII) [8], ICU length of stay (days), duration of mechanical ventilation (days), medications (type), sedation level using Richmond Agitation and Sedation Scale (RASS) [9], and delirium status (see below) were recorded. Data on redeemed prescriptions were obtained from population registers (Statistics Denmark) on all 942 patients eligible for the study and could only be linked to delirium status due to privacy. This provided us with an estimate of the usage of psychiatric medications prior to ICU-admission that might have affected the incidence of delirium.

### 3.3. Delirium Assessment

The Confusion Assessment Method for the ICU (CAM-ICU) was used to detect delirium during the ICU stay [10]. The CAM-ICU includes assessment of sedation level using RASS. Specially trained ICU nurses assessed the patients twice a day from ICU-admission to discharge. Additional assessments were made if the mental status of the patients changed noticeably. The delirium status was determined as “positive” if at least one CAM-ICU was positive during the ICU stay; otherwise “negative” if no CAM-ICU positive results or “unable to assess” (UTA) if patients were too deeply sedated to assess, or data were missing. The intensity of delirium was counted as number of positive assessments.

### 3.4. Posttraumatic Stress Disorder

The 16 PTSD items based on DSM-III-R in the fourth section of The Harvard Trauma Questionnaire (HTQ) were used in the present study [11]. Each item is rated ranging from “Not at all” (score = 1) to “Extremely” (score = 4). Total score is calculated as the sum of scores divided by the number of items answered; less than 2 indicates no PTSD-symptoms, 2 to 2.4 probable PTSD-symptoms, and higher than 2.4 positive PTSD-symptoms.

### 3.5. Anxiety

A short form of The State-Trait Anxiety Inventory (STAI) was used as the ten state anxiety questions were extracted: “Right now I feel… tense/nervous/restless/anxious/guilty/and so forth.” Answers were rated on a four-point Likert scale from “Not at all” (score = 1) to “Very much so” (score = 4). A sum score of 20 or more indicated clinically significant anxiety [12].

### 3.6. Depression

Major Depression Inventory (MDI) was used as a rating scale to measure the degree of depression [13] using 10 items to answer on a six-point Likert scale from “Never” (score = 0) to “Always” (score = 5) resulting in a total score ranging from 0 (no depression) to 50 (extreme depression). A total score less than 20 indicate no depression, 20–24: minor depression, 25–29: moderate depression, and 30 or more indicate severe depression. The MDI has been validated in the Danish population [13].

### 3.7. Memory

The ICU-Memory Tool (ICU-M) categorised memories in subscales as* factual memories* (family visit, alarms, voices, lights, faces, breathing tube, suctioning, darkness, clock, tube in nose, and ward rounds),* memories of feelings* (feeling uncomfortable, confused, down, anxious/frightened, in panic, and pain), and* memories of delusions* (hallucinations, nightmares, dreams, “feeling that someone was trying to hurt you”) [14]. We recorded the number of memories in each of the three categories. In a related study on the same cohort we found that delirium was associated with fewer factual and more memories of delusions [15]. In the present study we wished to explore the association between symptoms of PTSD and ICU-memories.

### 3.8. Health Related Quality of Life

Health related quality of life was assessed using the Short Form-36 (SF-36) providing information in eight specific domains: physical function, role physical, bodily pain, general health, vitality, social function, role emotional, and mental health [16].

### 3.9. Ethics

The study protocol was approved by the Danish Data Protection Agency and the National Health Service of Denmark. Approval was not required by the Regional Research Ethics Committee. No interventions were performed. Patients were advised of the voluntary nature of the study and their right to withdraw at any time.

### 3.10. Power

The number of participants included in each study was based on sample size calculations as established in a pilot study conducted in July 2009 and on patient characteristics in 2008. Our primary outcome was PTSD, and delirium the exposure. Assuming PTSD to have a prevalence of 22% [6] and delirium an incidence of 40% [5], an estimated relative risk of 1.5 had a power at 0.90 when 250 patients were included.

### 3.11. Data Analysis

We calculated the associations between post-ICU PTSD-symptoms, anxiety and depression, and the following.

- Age, gender, severity of illness, duration of mechanical ventilation, and ICU length of stay.
- ICU-memories of facts, feelings, and delusions.
- Health related quality of life.
- Antipsychotic medications administered in ICU.

Data are presented as proportions or as median and 10th and 90th percentile. Groups were compared using Chi-square test, Kruskal-Wallis test, or Wald test. The associations between number of delirium episode and scores at HTD, MDI, and STAI were estimated by linear regression. The associations between PTSD-symptoms, anxiety and depression, and number of memories were estimated by logistic regressions for each type of memories and adjusted only for age due to limited data. All analyses were done for both two and six month's data. Results were termed significant when *p* < 0.05. All analyses were performed in Stata/SE 13.1.

## 4. Results

### 4.1. Patient Characteristics

**Figure 1:**
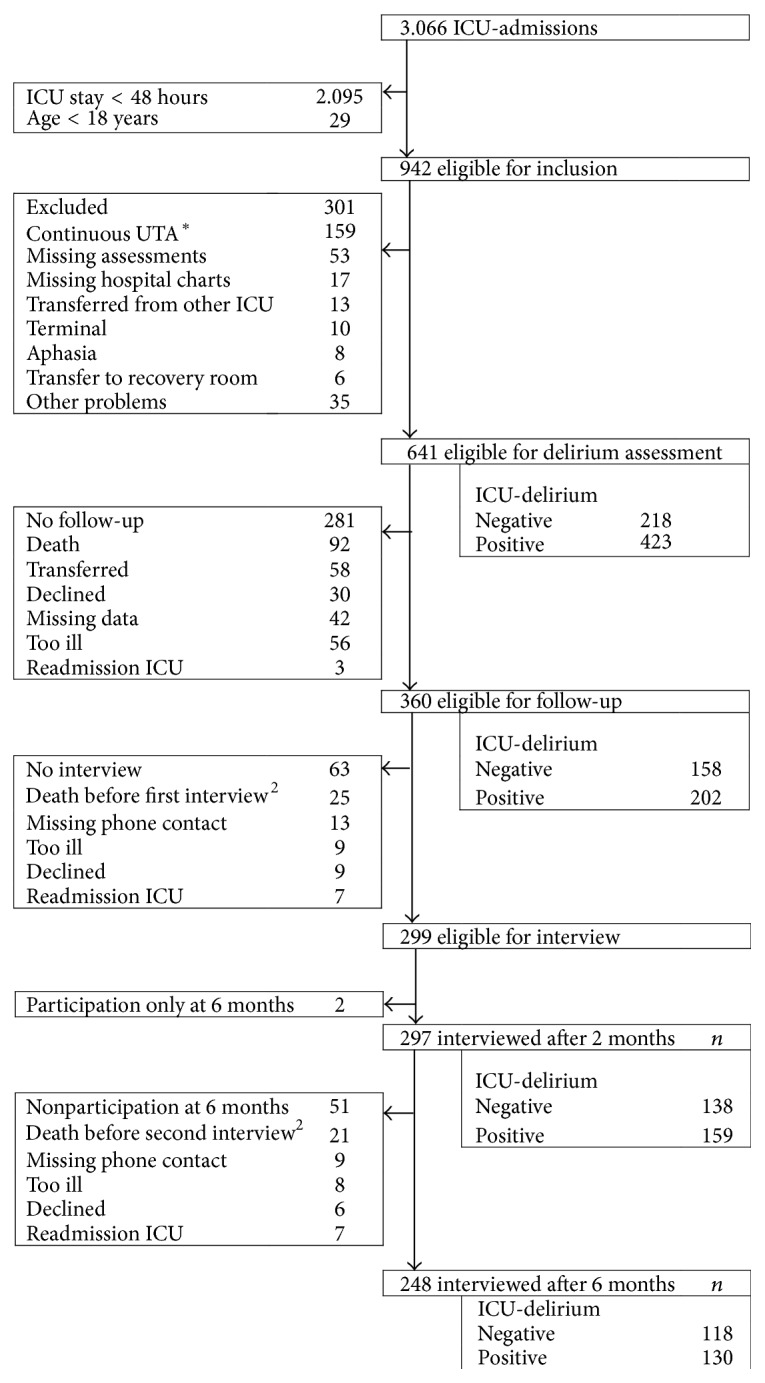
Cohort diagram of the study. ^*∗*^UTA: unable to assess (with the CAM-ICU). ^2^If patients were too ill and then died, only the first reason for nonparticipating is shown.

Information was provided for 641 eligible patients, 360 gave consent from January 2010 to Marts 2012, 297 were interviewed after 2 months, and 248 after 2 and 6 months (2 after 6 months only). The flow diagram in Figure 1 provides details on exclusion, inclusion and lost to follow-up. Interviewed patients were significantly younger, less severely ill at ICU-admission (SAPSII), mostly surgical patients had a shorter ICU stay, and fewer were delirious than patients that were not interviewed (Table 1).

### 4.2. Association between Delirium and Medications

Delirium was detected in more than half of the patients (*n* = 161, 54%) at least once during the ICU stay, and 138 of the patients were never delirious in the ICU (ever or never delirium). Due to the fluctuation of delirium this must be seen as a minimum incidence. A total of 189 patients (63%) were sedated for a mean of 3.84 days (95% CI 3.24; 4.42), and the remaining 110 patients were never sedated.

Preadmission mental illness was estimated by the number of redeemed prescriptions for psychotropic drugs. More delirious patients than nondelirious patients used antidepressants (11% versus 7%), anxiolytics (22% versus 18%), hypnotics (28% versus 23%), and antipsychotics (11% versus 7%) prior to ICU-admission. Although these findings were consistent, they were not statistically significant. Eight patients were medicated for Attention Deficit Hyperactivity Disorder (ADHD), five of these became delirious, and three were not assessable (UTA).

During the ICU stay, intravenous haloperidol was used in 29% of the 299 interviewed patients and in 53% of the 161 delirious patients. Interviewed patients received less haloperidol than noninterviewed, whereas the two groups used similar doses of other antipsychotics (Table 1). In 20% of the patients oral antipsychotics, mainly olanzapine, were used. Thirty-five patients (12%) received both haloperidol and other antipsychotics during the ICU stay. In nonresponders (*n* = 342) 23% received any antipsychotics.

### 4.3. Association between Delirium and PTSD-Symptoms, Anxiety, and Depression

We found no association between delirium and PTSD-symptoms, anxiety, or depression. OR (95% CI) at two months for PTSD was 1,25 (0,60; 2,57) anxiety 1,55 (0,59; 4,05) and depression 1,04 (0,72; 1,51). At six months OR for PTSD was 1,31 (0,63; 2,71), anxiety 1,14 (0,30; 4,35), and depression 1,04 (0,70; 1,53). For more details please see Table 2.

Symptoms of PTSD were found in 21 patients after 2 months, 12 of which had experienced delirium. At 6 months of follow-up, PTSD-symptoms were found in 12 patients (of which 8 were previously delirious) (Table 2, data for nonmissing).

Previously delirious patients were more anxious according to STAI after 2 months (12 versus 7), but after 6 months this was resolved, and 5 versus 4 had symptoms of anxiety (Table 2).

After two months, 30 patients suffered from depression according to the MDI; 12 (4%) had mild, 10 (4%) had moderate, and 8 (3%) had severe depression. In the severely depressed group, we found twice as many previously delirious compared to nondelirious patients (Table 2), though this was not statistically significant. Number of delirious episodes “severity of delirium” was not correlated with PTSD, anxiety, or depression neither at 2 or 6 months (data not shown).

When only considering the delirious patients, there were no differences in the psychometrics by delirium subtype (data not shown).

### 4.4. Association between Medications and PTSD-Symptoms, Anxiety, and Depression

Neither haloperidol nor sedatives and other psychotropic drugs significantly affected the prevalence of PTSD-symptoms, anxiety, and depression. At both interviews, no statistically significant difference was found between the prevalence of PTSD-symptoms, anxiety or depression, and SAPSII, admission type (medical/surgical), hospital site, length of ICU stay, days with delirium, or days with sedation. For ten of the 21 patients with HTQ scores indicating PTSD at two months, the STAI score showed anxiety, and eight of these had symptoms of depression at the MDI as well.

### 4.5. Association between Memories and PTSD-Symptoms, Anxiety, and Depression

The association of PTSD-symptoms, anxiety, and depression with memories of ICU depended on the type of memories experienced.* Memories of feelings* were significant in PTSD and depression outcomes at 2 months and at PTSD after 6 months, but insignificant after adjusting for age.

*Factual memories* were not associated with any of the outcomes.* Memories of delusions* were significant in anxiety after two months and remained so after adjusting as the only outcome. Of the participants, 93% had some kind of memory of the ICU. For further details see Table 3.

### 4.6. Association between Quality of Life and PTSD-Symptoms, Anxiety, and Depression

Based on answers from the 279 patients at the interview after 2 months and 240 patients after 6 months, there was a significant decrease in four domains of SF-36 (vitality, social function, role emotional, and mental health) if patients had PTSD-symptoms, anxiety, or depression. Bodily pain and general health (from SF-36) were reduced comparing patients with and without PTSD, anxiety, or depression. This was, however, not significant regarding PTSD-symptoms at 6 months (Table 4). Delirium was not found to affect the SF-36 scores (data not shown).

## 5. Discussion

Our study did not demonstrate an association between ICU-delirium and PTSD-symptoms or anxiety or depression at 2 and 6 months after discharge from ICU. We found that delirium in ICU was common (54%), and though the prevalence of depression, PTSD-symptoms, and anxiety was more common in post-ICU patients than the general population [12], it was maximum 10 percent. In this Danish cohort PTSD-symptoms, anxiety (according to the STAI), and depression were found in three times the known prevalence in the general Danish population. The frequency of these psychiatric disorders, did, however, not vary significantly between previously delirious and nondelirious patients in our study, in accordance with the findings of Girard et al. who also found that symptoms of PTSD were more frequent in the younger population [17].

Anxiety and depression are often present during the ICU stay and is significantly higher than in ward patients [18].

In a study from 1999 24% showed signs of anxiety and 14% had depression within 24 hours of ICU-admission in the group of patients who were able to communicate verbally [19]. However, they found only 7% with delirium, which might be explained by lack of assessment instruments. Though attention to delirium has risen the last 20 years, no change has been found in neuropsychological morbidities such as PTSD, anxiety, and depression [20], which can support that delirium does not lead to PTSD [21].

Memories of feelings or delusions in ICU affected PTSD-symptoms, anxiety, and depression, whereas factual memories did not. Most of the patients in our study had some recall of the stay in the ICU, in contrast to Jones et al. (2001), who found that most patients had no recall. Jones et al. concluded that ICU-memories might offer some protection against PTSD and anxiety, which is in accordance with our findings [22]. The disparate findings in the two studies might be explained by a trend toward lighter sedation in the past decade. The majority of our patients were nonsedated or only temporarily sedated. We assume that this might explain the fewer cases of PTSD-symptoms in our sample, as a mixed retrospective and prospective study demonstrated PTSD in 19% of the most sedated patients and absence of PTSD in the lightly sedated group with daily awakening [23]. A recent Danish study did, however, not find a difference in the rate of PTSD in lightly sedated patients with daily awakening compared to nonsedated patients [24]; other studies have indicated that PTSD is associated with deeper and longer sedation [25]. We can not determine if the low prevalence of PTSD can be influenced by less or no sedation, the open visiting time for relatives, or the sincerity about the delirium leading to less stress.

The local context in our ICU might have influenced the results of our study. The high nurse-patient ratio, free visiting hours, and absence of physical restraints might have had an impact on the incidence of delirium [26] as well as PTSD-symptoms. Physical restraints in other settings have been associated with prolonged sedation, development of PTSD, and recall of delusions [27].

The evidence on treatment of ICU-delirium is scarce. In our cohort, the administration of antipsychotic medications in the ICU was common; 29% received haloperidol and 20% received other psychopharmacological agents, often in combination. Based on three randomized studies, the National Institute for Health and Clinical Excellence in the United Kingdom (UK) [28] recommends starting at the lowest clinically appropriate dose and titrating cautiously according to symptoms. This is recommended despite the fact that haloperidol and olanzapine are not authorised for ICU-delirium treatment in the UK. Numerous studies on pharmacological management of delirious non-ICU patients are published [29]. Recommendations from these studies have been expanded to ICU patients, but one newer double-blind placebo-controlled randomized trial found that haloperidol did not modify delirium in critically ill patients, though a useful agent for management of agitation.

Our study showed that the four domains of mental health in SF-36 (vitality, social function, role emotional, and mental health) were decreased for patients with symptoms of PTSD, anxiety, or depression. It might be assumed that delirium was the cause as seen in other studies [30], but we found no association between ICU-delirium and reduced health related quality of life regardless of delirious memories [15]. Although our study failed to demonstrate an association between delirium and PTSD-symptoms, both syndromes need to be prevented.

### 5.1. Study Limitations

Participants in our study were younger, healthier, and less frequently delirious than nonparticipants indicating that we might underestimate the prevalence of later PTSD-symptoms. Since most nonparticipants failed to recover, we were unable to assess for PTSD-symptoms in this group. We did not aim to diagnose PTSD in our study but to detect symptoms of PTSD. A strong correlation, however, between PTSD and symptoms of PTSD was found in a self-reported questionnaire [31], and a Danish national population based study of the medical databases found a cumulative incidence risk for diagnosed psychometrics at 0.5 (95% CI 0.3–0.6) which support the low number PTSD, anxiety, and depression symptoms that we found [32].

Our choice of telephone interviews rather than face-to-face encounters was pragmatic. The method is less demanding for patients with post-ICU fatigue and improves participation. In contrast to questionnaires, telephone interviews afford participants to clarify questions and validate answers. Preexisting disease has been estimated as the most important factor on post-ICU health related quality of life [33], a factor we were unable to eliminate. One study has found that psychological interventions in ICU reduced symptoms of post-discharge PTSD [34]. In the same vein, the 1 : 1 nurse-patient ratio, open visiting hours, and lack of restraints in our setting might explain a reduced impact of delirium on long-term psychological sequelae.

## 6. Conclusions

Delirium was detected in 54% of our sample. Symptoms of PTSD were found in 8% of previously delirious patients after 2 months and in 6% after 6 months. Most patients (93%) had some recall of the ICU stay. We did not find an association between ICU-delirium and PTSD-symptoms, anxiety, and depression at 2 and 6 months after ICU-discharge. Memories of delusions were significantly associated with anxiety after two months. Remaining associations between types of ICU-memories and prevalence of post-discharge symptoms of PTSD, anxiety, and depression were insignificant after adjusting for age. PTSD-symptoms, anxiety, and depression were associated with a significant reduction in most domains of mental health in SF-36. We recommend more attention to delirium prevention, detection, and management in the ICU.

## Key Messages

Consider the following.

- ICU-delirium and post-discharge symptoms of PTSD anxiety or depression did not correlate.
- Memories of delusion in ICU affected anxiety after 2 months, whereas other psycometrics were not significant affected.

## Figures and Tables

**Table 1:** Demographics and outcomes.

	Interviewed	Not included	*p* value^#^
	*n* = 299^*∗*^	*n* = 342	
Age, median (10; 90 percentile)	62 (40; 78)	67 (46; 82)	<0.01
Gender			
Men, *n* (%)	166 (56)	244 (61)	0.12
Woman, *n* (%)	133 (44)	153 (39)	
SAPSII, median (10; 90 percentile)	34 (19; 59)	43 (24; 67)	<0.01
Type of admission			
Medical, *n* (%)	113 (38)	202 (51)	<0.01
Surgical, *n* (%)	186 (62)	195 (49)	
Delirium			
Negative, *n* (%)	138 (46)	80 (20)	<0.01
Positive, *n* (%)	161 (54)	262 (66)	
Unable to assess, *n* (%)	0 (0)	55 (14)	
IV haloperidol, *n* (%)	88 (29)	147 (37)	0.04
Atypical antipsychotics, *n* (%)	60 (20)	83 (21)	0.82
ICU stay, median (10; 90 percentile)	5 (2; 21)	6 (3; 25)	0.03

^*∗*^Attending either at two or six months or both. ^#^Chi-square test used for categorical data and Kruskal-Wallis test for numerical data. SAPSII = Simplified Acute Physiology Score. ICU = intensive care unit. IV = intravenous.

**Table 2:** Associations between delirium versus psychometrics.

	ICU-delirium	No-delirium	*p* ^#^
	*n* = 159	*n* = 138	

*After 2 months *			
PTSD			
None, *n* (%)	146 (92)	129 (93)	0.68
Present	12 (8)	9 (7)	
Probable, *n* (%)	9 (6)	8 (6)	
Severe, *n* (%)	3 (2)	1 (1)	
HTQ-score, median (10; 90 percentile)	1.14 (1.00; 1.89)	1.19 (1.00; 1.88)	
Anxiety			
None, *n* (%)	145 (92)	131 (95)	0.37
Present	12 (8)	7 (5)	
STAI-score, median (10; 90 percentile)	12 (10; 20)	12 (10; 18)	
Depression			
None, *n* (%)	142 (90)	124 (90)	0.95
Present	16 (10)	14 (10)	
Mild, *n* (%)	6 (4)	6 (4)	
Moderate, *n* (%)	5 (3)	5 (4)	
Severe, *n* (%)	5 (3)	3 (2)	
MDI-score, median (10; 90 percentile)	7 (1; 21)	6 (1; 20)	

	*n* = 130	*n* = 130	

*After 6 months *			
PTSD			
None, *n* (%)	121 (94)	114 (97)	0.44
Present	8 (6)	4 (3)	
Probable, *n* (%)	4 (3)	1 (1)	
Severe, *n* (%)	4 (3)	3 (3)	
HTQ-score, median (10; 90 percentile)	1.06 (1.00; 1.81)	1.06 (1.00; 1.63)	
Anxiety			
None, *n* (%)	125 (96)	114 (97)	0.85
Present	5 (4)	4 (3)	
STAI-score, median (10; 90 percentile)	11 (10; 17)	11 (10; 17)	
Depression			
None, *n* (%)	119 (92)	106 (90)	0.50
Present	11 (8)	12 (10)	
Mild, *n* (%)	3 (2)	6 (5)	
Moderate, *n* (%)	2 (2)	3 (3)	
Severe, *n* (%)	6 (5)	3 (3)	
MDI-score, median (10; 90 percentile)	4 (0; 18)	4 (0; 20)	

^#^Chi-square test comparing present to none. ICU = intensive care unit. PTSD = posttraumatic stress disorder. HTQ = Harvard Trauma Questionnaire. STAI = State-Trait Anxiety Inventory. MDI = Major Depression Inventory.

**Table 3:** The psychometrics associations to ICU-memories at ICU-Memory Tool.

	Memories of feelings		Memories of delusions		Memories of facts	
	OR (95% CI)	*p* ^#^	OR (95% CI)	*p* ^#^	OR (95% CI)	*p* ^#^
*After 2 months *						
PTSD	1.27 (0.99; 1.63)	0.06	1.06 (0.76; 1.48)	0.73	0.94 (0.83; 1.06)	0.32
PTSD^*∗*^	1.20 (0.92; 1.57)	0.17	1.00 (0.71; 1.40)	0.99	0.91 (0.80; 1.04)	0.16
Anxiety	1.29 (0.98; 1.68)	0.07	1.63 (1.16; 2.27)	<0.01	0.93 (0.82; 1.07)	0.32
Anxiety^*∗*^	1.20 (0.90; 1.60)	0.07	1.55 (1.10; 2.18)	0.01	0.90 (0.78; 1.04)	0.15
Depression	1.28 (1.03; 1.60)	0.03	1.01 (0.75; 1.36)	0.94	0.98 (0.88; 1.10)	0.77
Depression^*∗*^	1.17 (0.93; 1.48)	0.18	0.92 (0.67; 1.25)	0.59	0.95 (0.84; 1.06)	0.36

*After 6 months *						
PTSD	1.46 (1.06; 2.01)	0.02	1.43 (0.96; 2.14)	0.08	1.10 (0.91; 1.33)	0.31
PTSD^*∗*^	1.31 (0.94; 1.82)	0.11	1.26 (0.83; 1.91)	0.27	1.06 (0.88; 1.30)	0.51
Anxiety	1.02 (0.71; 1.46)	0.92	0.77 (0.41; 1.43)	0.41	0.94 (0.78; 1.13)	0.50
Anxiety^*∗*^	1.00 (0.68; 1.47)	0.97	0.75 (0.97; 1.40)	0.36	0.93 (0.77; 1.12)	0.44
Depression	1.08 (0.86; 1.36)	0.48	1.11 (0.80; 1.53)	0.54	1.03 (0.91; 1.17)	0.64
Depression^*∗*^	1.04 (0.82; 1.33)	0.75	1.06 (0.76; 1.48)	0.73	1.02 (0.89; 1.16)	0.80

^*∗*^Adjusted for age. ^#^Wald test. ICU = intensive care unit. PTSD = posttraumatic stress disorder.

OR = odds ratio. CI = confidence interval.

**Table 4:** The psychometrics associations to health related quality of life at the SF-36^*∗*^.

	*n*	Physical function	Role physical	Bodily pain	General health	Vitality	Social function	Role emotional	Mental health
*After 2 months *									
PTSD									
None	261	70 (10; 95)	25 (0; 100)	82 (25; 100)	62 (25; 92)	65 (25; 90)	100 (50; 100)	100 (33; 100)	88 (60; 100)
Present	18	58 (10; 90)	0 (0; 75)	37 (0; 100)	45 (15; 82)	35 (5; 55)	63 (0; 100)	33 (0; 100)	50 (28; 76)
*p* ^#^		0.21	0.04	<0.01	<0.01	<0.01	<0.01	<0.01	<0.01
Anxiety									
None	263	70 (10; 95)	25 (0; 100)	82 (25; 100)	62 (25; 92)	65 (25; 90)	100 (38; 100)	100 (33; 100)	88 (60; 100)
Present	16	50 (20; 80)	0 (0; 100)	42 (12; 100)	45 (15; 82)	43 (5; 80)	50 (13; 100)	33 (0; 100)	52 (20; 92)
*p* ^#^		0.07	0.04	<0.01	<0.01	<0.01	<0.01	<0.01	<0.01
Depression									
None	252	70 (10; 95)	25 (0; 100)	88 (34; 100)	64 (30; 92)	68 (30; 90)	100 (50; 100)	100 (67; 100)	88 (68; 100)
Present	27	45 (5; 90)	0 (0; 75)	37 (0; 100)	30 (10; 70)	30 (5; 50)	50 (13; 88)	33 (0; 100)	52 (32; 72)
*p* ^#^		0.02	<0.01	<0.01	<0.01	<0.01	<0.01	<0.01	<0.01

*After 6 months *									
PTSD									
None	230	75 (18; 100)	63 (0; 100)	82 (25; 100)	67 (25; 95)	70 (28; 90)	100 (50; 100)	100 (67; 100)	92 (62; 100)
Present	10	63 (15; 100)	0 (0; 100)	46 (18; 91)	46 (20; 86)	30 (5; 80)	56 (6; 100)	0 (0; 67)	58 (28; 82)
*p* ^#^		0.76	0.09	0.06	0.07	<0.01	<0.01	<0.01	<0.01
Anxiety									
None	234	75 (20; 100)	63 (0; 100)	82 (25; 100)	67 (25; 95)	70 (30; 90)	100 (50; 100)	100 (67; 100)	90 (64; 100)
Present	6	18 (0; 90)	38 (0; 100)	33 (0; 82)	39 (0; 67)	20 (0; 30)	50 (13; 50)	33 (0; 100)	54 (20; 68)
*p* ^#^		<0.01	0.30	<0.01	<0.01	<0.01	<0.01	<0.01	<0.01
Depression									
None	220	80 (20; 100)	75 (0; 100)	82 (25; 100)	67 (30; 95)	75 (35; 90)	100 (50; 100)	100 (84; 100)	92 (68; 100)
Present	20	53 (3; 93)	13 (0; 100)	25 (0; 82)	40 (5; 87)	20 (5; 85)	50 (19; 100)	33 (0; 100)	40 (18; 82)
*p* ^#^		0.01	0.01	<0.01	<0.01	<0.01	<0.01	<0.01	<0.01

^*∗*^Median (10; 90 percentile) at Short Form-36. ^#^Kruskal-Wallis test. ICU = intensive care unit. PTSD = posttraumatic stress disorder.

## Abbreviations

ADHD: Attention Deficit Hyperactivity Disorder
CAM-ICU: Confusion Assessment Method for the ICU
DSM: Diagnostic and Statistical Manual of Mental Disorders
HTQ: Harvard Trauma Questionnaire
ICU: Intensive care unit
ICU-M: ICU-Memory Tool
MDI: Major Depression Inventory
PTSD: Posttraumatic stress disorder
PTSS10: Posttraumatic stress syndrome 10-question inventory
RASS: Richmond Agitation and Sedation Scale
SAPSII: Simplified Acute Physiology Score
SF-36: Short Form-36
STAI: State-Trait Anxiety Inventory
UK: United Kingdom
UTA: Unable to assess.
